# The Anatomy of the Human Lung

**Published:** 1860-09

**Authors:** 


ART. X.—*The Anatomy of the Human Lung; an Essay for which was awarded the Fothergillian Gold Medal of the Medical Society of London.* By A. T. HOUGHTON WATERS, M.R.C.P., Lecturer, on Anatomy, Physiology, and Pathology in the Liverpool Royal Infirmary School of Medicine, etc. Demi 8vo., pp. 223. London: Churchill, 1860.

THE author of this valuable monograph has succeeded in placing before the profession the most complete and faithful exposition of the anatomy of the component structures of the human lung with which we are acquainted. Based upon numerous investigations and dissections of dead subjects, we may rely upon his statements as being accurate; and that portion of the work relating to the bronchial blood-vessels will be found to be especially interesting, inasmuch as the author has demonstrated the non-existence of bronchial veins within the lungs, a view in which he is seconded by Reisseisen. The Medical Society of London has done well in awarding the Fothergillian prize to Dr. Waters, who, by this work, has proved himself an original investigator and a careful observer. We regret that we cannot present our readers with an analytical notice of the work, as our space will only allow us to give the conclusions of the author:—

"The lungs consist of two portions: first, that which constitutes the *convective channels*, by means of which the air is carried to and from the true respiratory system; second, the *true respiratory portion*—that in which the process of respiration is carried on.

"The first portion is formed of the *trachea*, the *bronchi*, and the *bronchial tubes.*

"The second portion is formed of the *terminal dilatations of the bronchial tubes, together with a number of tubes given off from them*, to which the name of *air-sacs* has been given, to each assemblage of which the term *lobulette* has been applied; the terminal dilatation of the bronchial tube is, in fact, but the commencement of the lobulette, or the *point de reunion* of the various air-sacs.

"The *connective channels* consist of cartilaginous rings, muscular and fibrous tissue, and a mucous membrane which is lined by a columnar ciliated epithelium.

"The *respiratory portion* presents on the parietes of the parts of which it is composed a number of depressions, to which the term *alveoli* has been given; these *alveoli* exist in the air-sacs, and in terminal dilatation of the bronchial tubes, and in some animals in the ultimate bronchial tubes previous to their dilatation.

"The *air-sacs* and the *alveolated portion of the bronchial tubes* are lined by a variety of the pavement epithelium.

"The *pulmonary artery* distributes its blood to the respiratory portion of the lungs.

"The *pulmonary veins* return the blood which has been distributed by the pulmonary artery.

"The ultimate branches of the pulmonary artery in the air-sacs form the nutritious vessels of the respiratory portion of the lungs.

"The *bronchial arteries* distribute their blood to the bronchi, the bronchial tubes, the vessels and areolar tissue of the lungs; the branches that enter the lungs pour their contents into the pulmonary veins.

"The *bronchial veins* return the blood which is distributed to the structures about the roots of the lungs.

"The *lymphatic vessels* of the lungs form two sets, a superficial and deep. The former are found at the surface of the lungs; the latter chiefly accompany the bronchial tubes.

"The *nerves* of the lungs are derived from the pneumogastric and sympathetic; branches are distributed chiefly to the bronchial tubes, but some pass to the blood-vessels and to the surface of the lungs; some apparently are lost in the pulmonary tissue.

"The air-sacs exist fully developed in the fœtal lung before birth."

